# Enhanced Production of Aromatic Amino Acids in Tobacco Plants Leads to Increased Phenylpropanoid Metabolites and Tolerance to Stresses

**DOI:** 10.3389/fpls.2020.604349

**Published:** 2021-01-12

**Authors:** Moran Oliva, Aviv Guy, Gad Galili, Evgenia Dor, Ron Schweitzer, Rachel Amir, Yael Hacham

**Affiliations:** ^1^Department of Plant and Environmental Sciences, Weizmann Institute of Science, Rehovot, Israel; ^2^Laboratory of Plant Science, MIGAL – Galilee Research Institute, Kiryat Shmona, Israel; ^3^Tel-Hai College, Upper Galilee, Israel; ^4^Department of Weed Research, Agriculture Research Organization, Newe Ya’ar Research Center, The Volcani Center, Ramat Yishay, Israel

**Keywords:** aromatic amino-acids, aroG, parasitic plant, *Phelipanche aegyptiaca*, salt stress, shikimate pathway, tobacco (*Nicotiana tabacum*)

## Abstract

Aromatic amino acids (AAAs) synthesized in plants via the shikimate pathway can serve as precursors for a wide range of secondary metabolites that are important for plant defense. The goals of the current study were to test the effect of increased AAAs on primary and secondary metabolic profiles and to reveal whether these plants are more tolerant to abiotic stresses (oxidative, drought and salt) and to *Phelipanche egyptiaca* (Egyptian broomrape), an obligate parasitic plant. To this end, tobacco (*Nicotiana tabacum*) plants were transformed with a bacterial gene (AroG) encode to feedback-insensitive 3-deoxy-D-arabino-heptulosonate 7-phosphate synthase, the first enzyme of the shikimate pathway. Two sets of transgenic plants were obtained: the first had low expression of the AroG protein, a normal phenotype and minor metabolic changes; the second had high accumulation of the AroG protein with normal, or deleterious morphological changes having a dramatic shift in plant metabolism. Metabolic profiling analysis revealed that the leaves of the transgenic plants had increased levels of phenylalanine (up to 43-fold), tyrosine (up to 24-fold) and tryptophan (up to 10-fold) compared to control plants having an empty vector (EV) and wild type (WT) plants. The significant increase in phenylalanine was accompanied by higher levels of metabolites that belong to the phenylpropanoid pathway. AroG plants showed improved tolerance to salt stress but not to oxidative or drought stress. The most significant improved tolerance was to *P. aegyptiaca*. Unlike WT/EV plants that were heavily infected by the parasite, the transgenic AroG plants strongly inhibited *P. aegyptiaca* development, and only a few stems of the parasite appeared above the soil. This delayed development of *P. aegyptiaca* could be the result of higher accumulation of several phenylpropanoids in the transgenic AroG plants and in *P. aegyptiaca*, that apparently affected its growth. These findings indicate that high levels of AAAs and their related metabolites have the potential of controlling the development of parasitic plants.

## Introduction

The shikimate pathway is initiated by combining phosphoenolpyruvate (PEP) and erythrose-4-phosphate (E4P) with the activity of 3-deoxy-D-arabino-heptulosonate 7-phosphate synthase (DAHPS). Following six enzymatic steps, it ends with the production of chorismate, which is then used to generate three aromatic amino acids (AAAs): tryptophan, phenylalanine, and tyrosine (Yoo et al., 2013). These AAAs are used for protein synthesis and for the synthesis of different secondary metabolites that play a major role in protecting plants from biotic and abiotic stresses (Supplementary Figure S1).

Studies have shown that the levels of AAAs are mainly regulated by: (i) the expression levels of genes encoding enzymes in the pathway that are controlled by both developmental and environmental signals (reviewed by Maeda and Dudareva, 2012); (ii) the levels of precursors of the shikimate pathway (PEP and E4P) (Maeda and Dudareva, 2012); (iii) the rate of AAAs catabolism that leads to biosynthesis of their downstream metabolites (Galili et al., 2016); and (iv) the activity of key enzymes in the shikimate and AAAs pathways, namely, DAHPS, chorismate mutase (CM), arogenate dehydratase (ADT), anthranilate synthate (AS), and arogenate dehydrogenase (ADH) that were shown to be regulated allosterically by products of the pathway (Li and Last, 1996; Huang et al., 2010; Tzin and Galili, 2010; Westfall et al., 2014; Supplementary Figure S1).

Studies have also shown that under abiotic stresses, the expression levels of genes in the AAAs biosynthesis pathway are up-regulated, leading to higher levels of AAAs and their secondary metabolites, including flavonoids, anthocyanins, betalains, and phenolic acids (reviewed by Tzin and Galili, 2010; Maeda and Dudareva, 2012). Indeed, abiotic stresses such as drought, heavy metal, salinity, high/low temperature, ultraviolet radiation, nutrient deficiencies, wounding, pathogen attacks, and herbicide treatment promote the phenylpropanoids pathway that produced most of these metabolites (Solecka, 1997; Sharma et al., 2012). In addition, high levels of AAAs and their related secondary metabolites were associated with a high tolerance ability of plants to biotic and abiotic stresses (Maeda and Dudareva, 2012; Sharma et al., 2019; Francini et al., 2019). Moreover, salicylate, a product of the shikimate pathway, is involved in the adaptation of plants to the stress conditions (Loake and Grant, 2007; Khan et al., 2015). Thus, the induction of AAAs could potentially contribute directly and indirectly to the stress tolerance of the plants.

A mutant of bacterial DAHPS enzyme (called AroG) that has a substitute of leucine at position 175 with glutamine (AroG_175_) led to the production of phenylalanine feedback-insensitive enzyme (Tzin et al., 2012). Overexpression of this gene led to significantly higher levels of AAAs and their specific secondary metabolites in transgenic *Arabidopsis thaliana*, purple Petunia hybrida V26, tomato *(Solanum lycopersicum)* fruits and grape (*Vitis vinifera*) cell culture (Tzin et al., 2012, 2013; Manela et al., 2015; Oliva et al., 2015).

The objectives of the current study were to study the effect of AroG overexpression on the phenotype of tobacco (*Nicotiana tabacum*) plants and on their metabolic profiling, and to test the ability of AroG transgenic plants in coping with abiotic stresses (salt, drought, and oxidative) and biotic stresses caused by the parasitic plant, *Phelipanche aegyptiaca*.

## Materials and Methods

### Plasmid Construction, Plant Transformation, and Growth

The feedback-insensitive AroG_175_ construct that was used previously to transform *A. thaliana*, petunia, tomato, and grape cells (Tzin et al., 2012, 2013; Manela et al., 2015; Oliva et al., 2015) was used to transform tobacco WT (*Nicotianata tobacum* cv Samsun NN) plants (Supplementary Figure S2). Control plants having an empty vector (EV) were generated by transforming WT plants with the plasmid used for AroG cloning (pBART; Tzin et al., 2012) that contain only the kanamycin-resistant gene. For the plant transformation, sterile tobacco seeds were grown on Nitsch media (Duchefa, Netherlands) with 2% (w/v) sucrose in a growth chamber under a 16/8 h light/dark cycle at 25°C. After 4 weeks, leaves from the sterile tobacco plants were transformed, as previously described (Horsch et al., 1985). Transgenic plants were selected on Nitsch media containing 100 mg L^–1^ kanamycin and 400 mg L^–1^ carbenicillin, and the kanamycin-resistant plants were transferred to soil for further growth. To obtained propagate plants, T_0_ transgenic AroG and control plants (WT and EV) were propagated from leaves using 2 μM benzyl adenine (BA) and 0.01 μM indole acetic acid (IAA) in MS medium with 3% sucrose. After 4 weeks, developing shoots were removed from the calli to allow rooting in MS medium with 150 mg/L kanamycin. The developing shoots from WT plants were transferred to MS medium without antibiotics. Rooted shoots of similar size and having a similar root system were transferred to soil in 1L pots and were grown in a growth chamber under a 16/8 h light/dark cycle at 25 ± 3°C. For the abiotic stress, 1L pots were grown in a greenhouse during the spring with 20–28°C with no artificial light.

### Quantitative Real-Time PCR and Immunoblot Analyses

The protein expression levels of the bacterial AroG were measured in the leaves of 10-week-old transgenic AroG plants that were resistant to kanamycin and in the leaves of WT and EV using immunoblot and antibodies against the three copies of hemagglutinin (3xHA) epitope tag that fused to the AroG gene (Supplementary Figure S2). The transcript expression level was measured by quantitative real-time PCR (qRT-PCR), as previously described (Matityahu et al., 2013). Primers used for (qRT-PCR) analyses are listed in Supplementary Table S2.

### Extraction and Analysis of Primary Metabolites Using Gas Chromatography-Mass Spectrometry (GC-MS)

Primary metabolites were extracted from 20 mg of dried powder from the leaves of the tobacco AroG and the control WT and EV plants. The extraction and derivatization method was employed using norleucine as internal standard (0.2 mg mL^–1^), as previously described (Cohen et al., 2016). Volumes of 1 μL were injected into the GC-MS column. A set of retention time standards consisting of alkanes of increasing molecular mass dissolved in pyridine were injected after each set of 10 samples. Metabolites were detected using GC-MS, as previously described (Malitsky et al., 2008; Mintz-Oron et al., 2008). The Xcalibur software v.3.1 (Thermo Finnigan) was used for data analysis, and compounds were identified by comparing their retention index (RI) and mass spectrum to those generated by standards and analyzed using the same column and under similar conditions. When standards were not available, compounds were identified putatively by comparing their RI and mass spectrum to those present in the mass spectra GMD VAR5 library of the Max Planck Institute for Plant Physiology, Golm, Germany, and the commercial mass spectra library NIST05^1^.

### Total Phenolic Compounds Content Determination

For total phenolic compounds content determination, 20 mg dried powder from leaves were ground in 0.5 mL water, and the colorimetric method (Ben Nasr et al., 1996) was employed. Ten microliter of the extraction sample were loaded on a 96 well ELISA plate. To each well, 50 μL of 10% Folin-Ciocalteu reagent and 40 μL of 7.5% (w/v) Na_2_CO_3_ were added. The plate was incubated for 40 min at 37°C and then read at 765 nm (Infinite M200PRO, Tecan, Grodig, Austria). A standard curve was created using gallic acid in a range of 0.5–10 μg.

### Detection of Phenolic Compounds in Tobacco Leaves Using uHPLC-DAD

Twenty mg of dry tissue were ground in 1 mL distilled water. The extraction was shaken for 40 min at 50°C, then centrifuged for 20 min at 20,000 g. The supernatant was filtered through 0.45 μm filters (Millipore) and kept frozen at −70°C until used. The chromatographic analysis was performed using UltiMate 3000 modules consisting of a solvent delivery module LG-980-02, pump LPG-3400SD, autosampler WP53000TSL, column compartment TCC3000SD and detector DAD3000 UV/vis (Ultimate 3000, Thermo Fisher Scientific). Ten microliter from the water extraction were injected into a Hydro-RP-C18 Synergi column (3 mm × 100 mm, particle size = 2.5 μm, Phenomenex, United Kingdom). Elution was performed at a flow rate of 0.8 mL/min using a mixture of water/Formic acid (99:1 v/v) (A) and acetonitrile (B) as a mobile phase. The run time was 35 min, and the samples were eluted by the following gradient: 100% A and 0% B at time 0, followed by 96% A and 4% B at 3.6 min, 85% A and 15% B for 22 min, 50% A and 50% B at 23 min, 20% A and 80% B from 25 to 28 min, 100% A and 0% B at 32 min until 35 min. Detection was performed at 280 nm.

### Detection of Phenylpropanoids Compound in *P. aegyptiaca* and Tobacco Roots Using HPLC and LC-MS/MS Analysis

For phenylpropanoids determination by HPLC, 20 mg of dry tissue were extracted in 80% methanol. The samples were analyzed by injecting 5 μL of the extracted solutions into an uHPLC connected to a photodiode array detector (Dionex Ultimate 3000) with a reverse-phase column (Phenomenex RP-18, 100 mm, 3.0 mm, 2.5 μm). The mobile phase consisted of (A) DDW containing 0.1% formic acid and (B) acetonitrile containing 0.1% formic acid. The gradient started with 5% B for 2 min, then increased to 98% B in 20 min and maintained at 98% B for another 3 min. Phase B was returned to 5% for 2 min and the column was allowed to equilibrate at 5% B for 5 min before the next injection. The flow rate was 0.4 mL/min. The LC-MS/MS analysis was performed with a Heated Electrospray ionization (HESI-II) source connected to a Q Exactive^TM^ Plus Hybrid Quadrupole-Orbitrap^TM^ Mass Spectrometer Thermo Scientific^TM^. ESI capillary voltage was set to 3,500 V, capillary temperature to 300°C, gas temperature to 350°C and gas flow to 35 mL/min. The mass spectra (m/z 100–1,000) were acquired in both negative-ion and positive-ion modes with high resolution (FWHM = 70,000). For MS^2^ analysis, the collision energy was set to 15, 50 and 100 EV. For data preprocessing, the peak area integration was performed with Compound Discoverer 3.1 (Thermo Fisher Scientific, Version 3.1.0.305). Several of the compounds, vanillin, ferulic acid, cinnamic acid, chlorogenic acid, caffeic acid, and 4-coumaric acid, were identified based on standards (all standards were purchased from Sigma, except for trans-ferulic acid, which was purchased from TCI, Tokyo Chemical Industry). Other compounds were identified based on the MzCloud database using MS^2^ data and the ChemSpider database using HRMS (Supplementary Table S4).

### Abiotic Tolerance Assays

For the salt tolerance examination, the propagated plants having 6–8 leaves were acclimated in a greenhouse for 2 weeks, and plants of similar size were used for the stress experiments. The experiments were conducted under greenhouse conditions at 20–28°C. The plants were irrigated every 2 days with 250 mL of 150 mM NaCl solution for 21 days. After 21 days of salt stress, the height (cm), total dry biomass (mg) of aerial parts, senescence level (number of yellow and senescing leaves out of total leaves number) and plant vigor score were measured. The vigor score is based on phenotype, whereby “1” is the score for plants having wilting and senescing leaves that were out of full turgor, and “9” is the score for plants that look normal with full turgor. Each line tested was compared to control EV plants under the same stress conditions.

For the oxidative stress assay, 8 mm of discs from the leaves of T_0_ propagated transgenic and control plants that were grown in a greenhouse were placed in plates with 0, 1, 2, 5 μM methyl viologen (paraquate) under 80–110 μE light conditions for 20 h. The chlorophyll content of the leaf discs was then measured using ImageJ software^2^. Change was calculated and marked as green level (in%) compared to control (distilled water).

For the drought stress assay, propagated plants were moved to 1L posts in a greenhouse and were irrigated normally for 3 weeks (six developed leaves). To generate the first period of drought stress, irrigation was stopped for 10 days. After 10 days of drought when early morning wilting was observed, senescence level and plant vigor score were measured. The plants were then watered fully for 3 days to full recovery. Followed recovery, a second drought period was applied for 10 days. At the end of the second drought, parameters of height, dry biomass and relative water content (RWC) were measured. For relative water content (RWC), leaf samples (8 mm discs) were floated overnight in deionized water. Turgid weight was determined, and all of the samples were dried at a constant temperature. Dry weight was measured and percent relative water content (RWC,%) was calculated 100^∗^ (fresh weight – dry weight/turgid weight – dry weight). RWC was measured with 4–8 biological repetitions.

### *P. aegyptiaca* Tolerance Experiments

Homozygous seeds of WT, EV, AroG lines 2.1 and 3.1 were germinated and grown on Nitsh media (Duchefa) for 3 weeks. Seedlings were transferred to 2-L pots (Tefen Nachsholim, Israel) using medium heavy clay-loam soil containing a dry weight basis of 55% clay, 23% silt, 20% sand, 2% organic matter, pH 7.1 (one plant per pot). Slow-release fertilizer at a concentration of 0.6% (w/v) (Osmocote, Scotts Miracle-Gro, Marysville, OH) and *P. aegyptiaca* seeds at a concentration of 15 ppm (15 mg seeds kg^–1^ soil ∼2,250 seeds kg^–1^) were added to the soil (Hacham et al., 2016). The above-mentioned components were mixed to homogeneity in a cement mixer for 10 min. The pots were placed in a net house and drip-irrigated. When the tobacco plants were fully developed and flowered, the shoots were cut at the soil surface. The roots were washed gently, removing the soil under tap water, and dried on a paper towel. *P. aegyptiaca* at all developmental stages were collected from each plant, and their numbers and biomasses were recorded. The biomass of the tobacco roots and shoots were also measured.

### Statistical Analyses

The data represent the mean of the independent replicates. Statistical significance was evaluated using JMP software version 8.0 (SAS Institute Inc., Cary, NC). Significant differences between treatments were calculated according to the Tukey-Kramer HSD test or the student t-test (*p* < 0.05). Principal component analysis (PCA) was conducted using the MetaboAnalyst 3.0 comprehensive tool^3^ (Xia et al., 2015) with auto scaling manipulations. Graphs were compiled using GraphPad Prism 5.01 scientific software^4^.

## Results

### Several Tobacco Plants Having a High Expression of the *E. coli* Feedback-Insensitive AroG_175_ Exhibited a Severe Abnormal Phenotype

**FIGURE 1:**
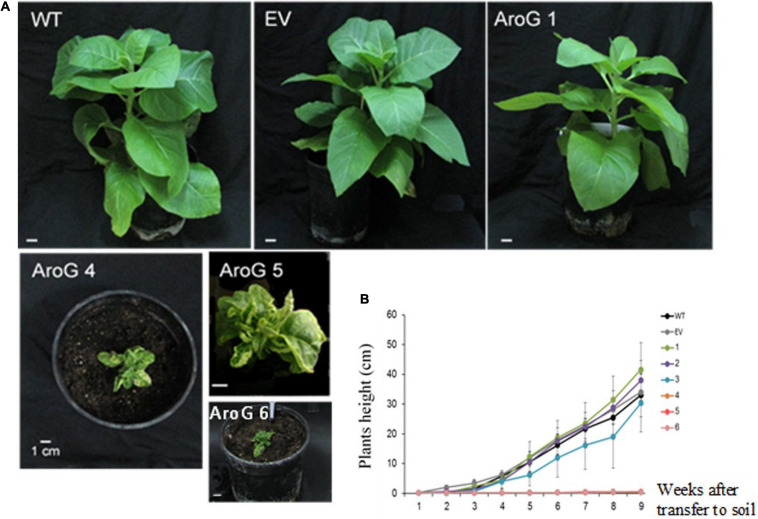
Phenotype of transgenic plants expressing the bacterial AroG gene. **(A)** The photos were taken 9 weeks after transferring to soil. **(B)** Growth rate measurements. Four-week propagated plants were transferred to soil; shoot height was measured every 7 days for a period of nine additional weeks. Data are presented as mean ± SE (*n* = 4–7). WT, wild type plants; EV, transgenic plants having an empty vector; AroG, plants overexpressing the bacterial AroG gene; that had a normal phenotype (lines #1–3) and an abnormal phenotype (lines #4–6).

**FIGURE 2:**
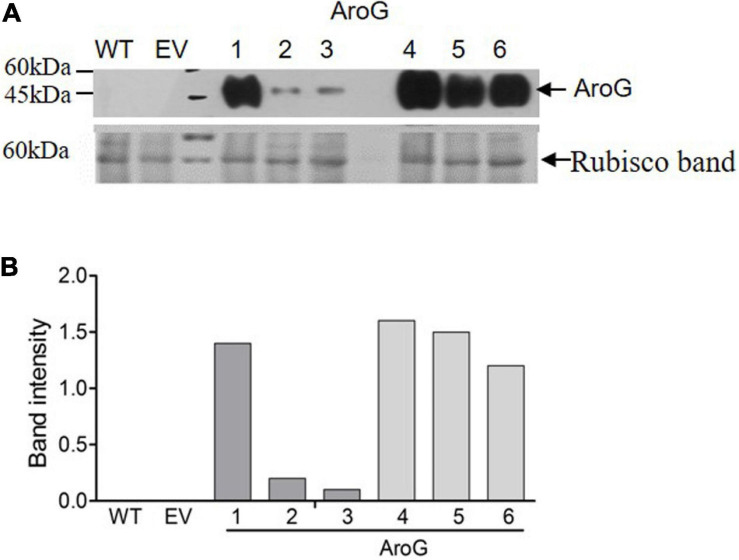
Expression level of bacterial AroG in transgenic tobacco plants. **(A)** Immunoblot analysis of protein extracts from the leaves of WT, EV and transgenic plants expressing AroG using anti-HA antibodies. Lower panel: coomassie brilliant blue staining used for equal loading; the rubisco band is marked by an arrow. The marker size is shown on the left. **(B)** The graph represents band intensity obtained from the immunoblot analysis that was normalized to the rubisco band as measured by ImagJ.

To study the impact of high AAAs levels on primary metabolic profile and on the ability of tobacco plants to cope with stresses, we overexpressed the *E. coli* AroG_175_ gene targeted to the chloroplast under the control of the 35S CaMV promoter and octopine synthase terminator (Tzin et al., 2012; Supplementary Figure S2). Thirty kanamycin-resistant T_0_ tobacco lines were screened for expression of the AroG gene by immunoblot analysis using antibodies against the 3HA epitope-tag that was fused to the AroG gene (Supplementary Figure S2). Out of the 30 lines, 22 plants showed a band of the expected 42 kDa polypeptide (Supplementary Figure S3). Fifteen plants exhibited a morphological phenotype similar to the empty vector (EV) and wild type (WT) plants that were used as controls. However, seven transgenic lines (Supplementary Figure S3) exhibited a severe abnormal phenotype, including slow developmental rate, failure to produce flowers, narrow curled pale leaves and loss of apical dominance (Figure 1A). Based on these phenotypes we chose for further analysis, three lines (marked as lines #1–3) that exhibited a normal phenotype and three lines (marked as lines #4–6) that had abnormal phenotypes compared to the EV and WT plants. Due to the inability of the plants having the abnormal phenotype to produce flowers, we propagated the plants using a tissue culture and generated shoots from the leaves of T_0_ AroG transgenic plants and from EV and WT plants. The regenerated plants exhibited a phenotype similar to their original transgenic lines. The growth rate of the different propagated plants was measured to show that lines #4–6 have severe growth retardation compared to lines #1–3 and the control plants (Figure 1B). To reveal the relationship between the morphological phenotype of the transgenic plants to the AroG protein accumulation, immunoblot analysis was made for comparison of the six selected lines. The immunoblot analysis showed that transgenic lines #4–6 had high protein accumulation, about 7- to 10-fold compared to lines #2–3 (Figure 2). Notably, line #1 that showed a high expression level of protein exhibited a normal morphological phenotype.

The severe abnormal phenotype of lines #4–6 could be attributed to the high auxin levels produced from tryptophan. To test this assumption, the expressions levels of two auxin response genes were measured using qRT-PCR, but the changes were insignificant compared to those having a normal phenotype. Moreover, feeding experiments carried out on WT plants having different auxin contents did not suggest that the phenotype was due to the higher auxin content (see Supplementary Text S4 for more details; Supplementary Figures S4,S5).

### The Levels of Aromatic Amino Acids and Their Associated Metabolites Increased in the Transgenic Plants

**FIGURE 3:**
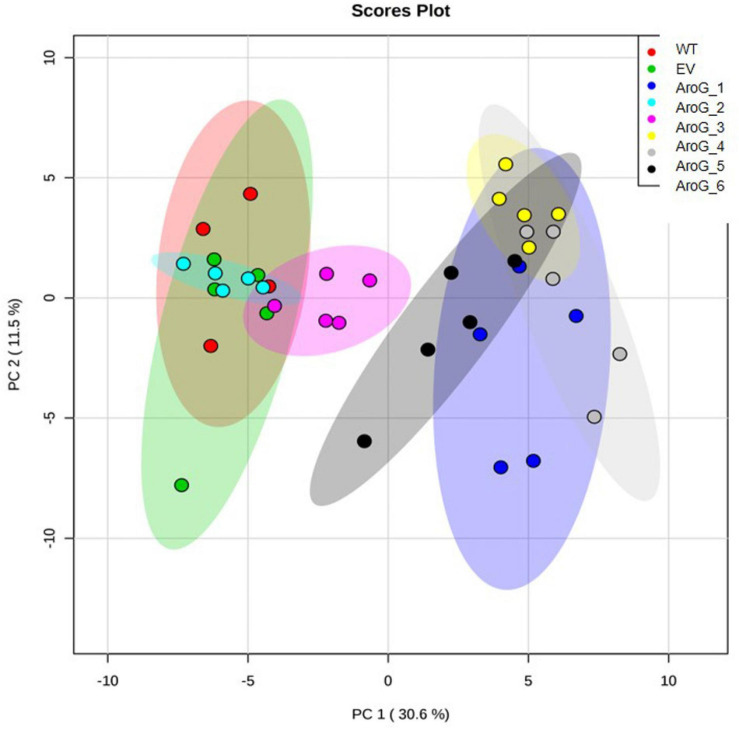
PCA plot of GC-MS data set obtained from the leaves of WT, EV, and transgenic AroG lines #1–6. Variances explained by the first two components (PC1 and PC2) appear in parentheses. WT (*n* = 4), EV and AroG plants (*n* = 5).

**FIGURE 4:**
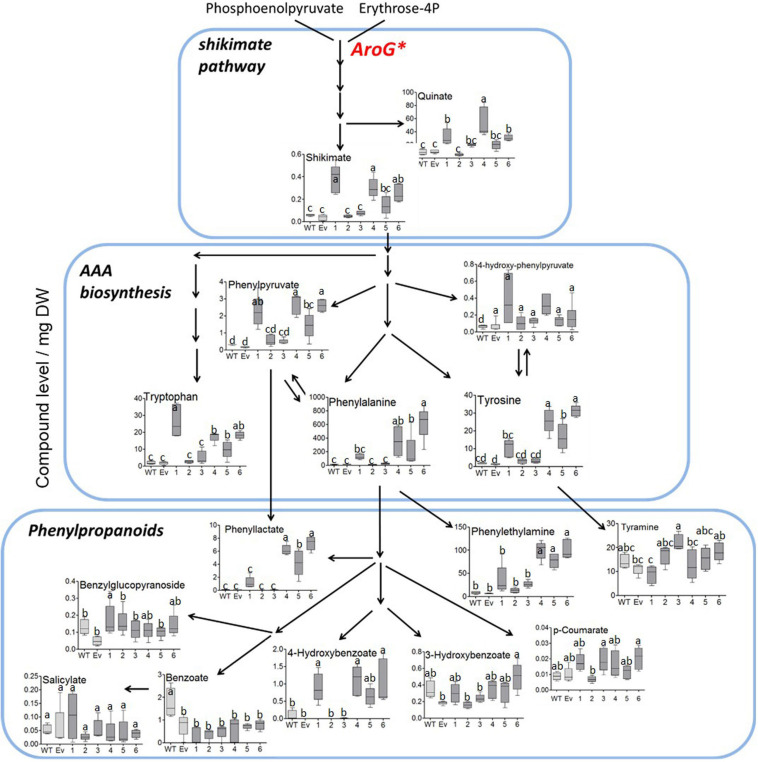
Levels of AAAs and their associated metabolites as detected by GC-MS in the leaves of WT (*n* = 4), EV (*n* = 5) and transgenic tobacco AroG lines #1–6 (*n* = 5). Results are presented on a pathway scheme with the graph representing compound level as the peak area normalized to the internal standard (norleucine) and calculated for 1 mg of dry weight (DW).

To study the effect of higher expression levels of AroG on plant metabolic profiling, GC-MS analysis was used on extracts from the leaves of 10-week-old propagated tobacco plants. Seventy-six metabolites were detected using GC-MS (Supplementary Table S1). Principal component analysis (PCA) showed that lines #2–3 having a low AroG protein accumulation were grouped close to the control plants (WT/EV), while lines #1, #4–6 were grouped separately (Figure 3). The observation that line #1 was grouped together with lines #4–6, which exhibited an abnormal phenotype, suggested that a high accumulation of AroG protein resulted in a significantly altered metabolic profile. These four lines had significantly higher levels of AAAs compared to WT/EV. The level of phenylalanine increased up to 43-fold, tyrosine up to 24-fold and tryptophan up to 10-fold compared to EV (Figure 4 and Supplementary Table S1). Lines #2–3 had slightly increased levels of AAAs, but with no significant difference compared to EV (Figure 4 and Supplementary Table S1). In addition to AAAs, the levels of shikimate, quinate, and phenylpyruvate (that related to the AAA pathway), significantly increased in lines #1, #4–6 compared to WT/EV (Figure 4 and Supplementary Table S1). The levels of phenylalanine downstream metabolite, 4-hydroxybenzoate, also increased significantly in these lines (Figure 4 and Supplementary Table S1), while phenyllactate and phenylethylamine increased significantly only in lines #4–6, which exhibited an abnormal phenotype (Figure 4 and Supplementary Table S1).

**FIGURE 5:**
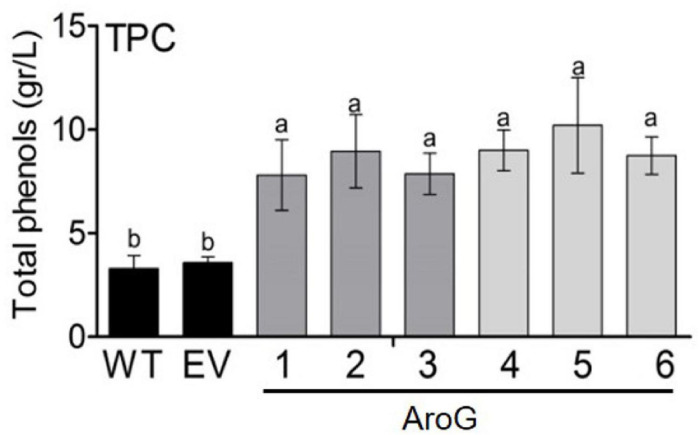
Total polyphenol content (TPC) in the leaves of transgenic plants overexpressing the bacterial AroG gene and EV/WT. TPC is represented as g gallic acid equivalents per liter of water extract. Data are presented as the mean ± SE of four biological repetitions. Different letters represent statistical significance (*p* ≤ 0.05) which was determined using the Tukey-Kramer test.

Since the shikimate pathway contributes to the production of phenolic compounds in plants, total phenolic content (TPC) was measured in young leaves. All of the transgenic lines had higher TPC by about 2- to 2.8-fold compared to EV, but with no significant difference between transgenic lines having high AroG expression (lines #1, #4–6) and those with low expression (lines #2, #3) (Figure 5). The similar TPC level in the different AroG lines was unexpected since these lines differ at the levels of AAAs and their associated metabolites. Therefore, we examined the differences between profiles of the phenols using HPLC-DAD at 280 nm. As was shown for the TPC, the HPLC-DAD analysis revealed that AroG lines had higher levels of phenols compared to WT/EV. However, this analysis showed that different phenols increased in the different AroG lines. Lines #4–6 had higher levels of phenols no. 1, 4 and 8, lines #2 and #3 had higher levels of phenol no. 5, and lines #1 and #2 had higher levels of phenol 2 (see Supplementary Text S6 for more detail; Supplementary Figure S6).

### The Levels of Most of the Primary Metabolites Did Not Change Significantly in the Transgenic Plants

**FIGURE 6:**
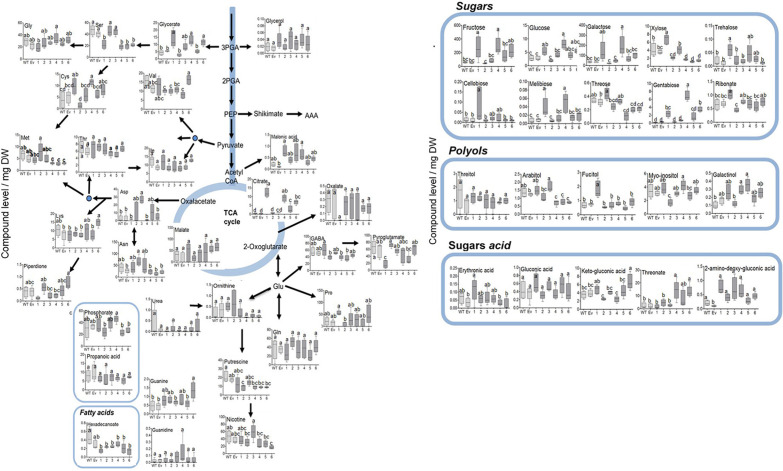
Primary metabolites profile of plants overexpressing the bacterial AroG gene. Metabolites analysis was performed using GC-MS in the leaves of WT (*n* = 4), EV (*n* = 5), and transgenic tobacco AroG lines #1–6 (*n* = 5). Data represent compound level as the peak area normalized to the internal standard (norleucine) and calculated for 1 mg of dry weight (DW).

Since the shikimate pathway and its associated metabolites used about 30% of the photo- assimilate carbon (Maeda and Dudareva, 2012; Galili et al., 2016), we next studied the effect of AroG expression on a wide range of primary metabolites. GC-MS analysis revealed that the changes in AAAs levels in the transgenic lines did not affect most of the other amino acids, with the exception of serine that decreased in lines #1, #4–6, methionine that decreased in lines #4–6, aspartate that increased in line #3, cysteine that increased in lines #1 and #4, and lysine that increased in line #6 (Figure 6 and Supplementary Table S1).

In lines #1, #4–6 having a high AroG protein level, several differences in sugars levels were observed. The levels of threose and xylose were reduced in lines #4–6 compared to WT/EV, while the levels of the other sugars increased, including fructose, glucose, melibiose, β-D-galactopyranoside (in lines #1 and #4), trehalsoe (in lines #1 and #3) and gentabiose (in lines #1, #4 and #6) (Figure 6 and Supplementary Table S1). An increase was also detected in sugar acids, threonate (in lines #4 and #6) and glycerate (in lines #1, #4, and #6) (Figure 6 and Supplementary Table S1). Citrate increased significantly in lines #1, #4–6 while other TCA intermediates didn’t change significantly compared to WT/EV (Figure 6 and Supplementary Table S1). The results of the primary metabolites analysis imply that the induction of the shikimate pathway by a high expression of the AroG protein resulted in mild changes in the plants’ primary metabolism. Most of the changes were detected in the sugars content.

### The Effect of a High Expression Level of AroG on the Ability of the Plants to Cope With Abiotic Stresses

Studies have shown that in response to environmental stresses, plants induce the shikimate pathway and produce higher levels of AAAs and phenolic metabolites (Dixon and Paiva, 1995; Dixon, 2001; Francini et al., 2019; You et al., 2019). However, it is not yet clear if plants having higher levels of these metabolites can be more tolerant to abiotic stress. To obtain more knowledge on this possibility, tobacco lines #1–3, which had higher TPC and a normal phenotype, were tested for their abilities to cope with salt, oxidative and drought stresses.

**FIGURE 7:**
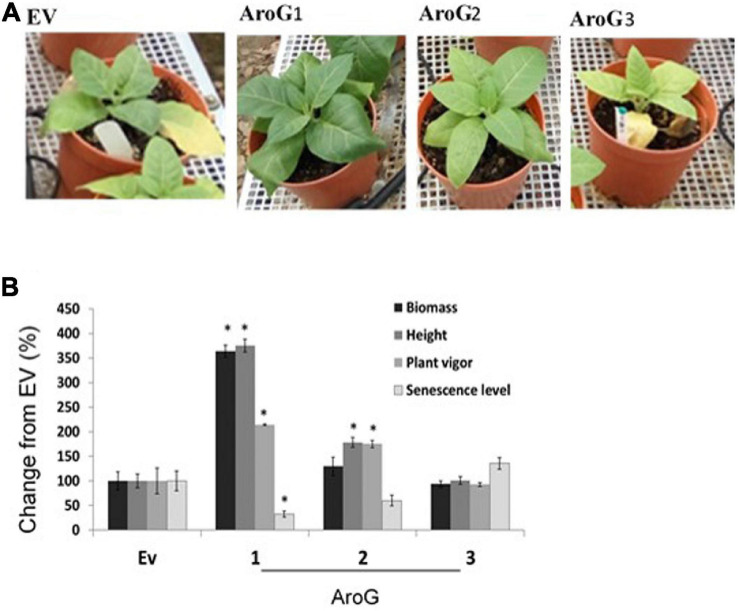
The ability of the transgenic plants overexpressing the bacterial AroG to cope with salt stress. **(A)** The phenotype of representative plants from each group 21 days after the salt-stress (150 mM) period. **(B)** The graph shows the% changes in different growth parameters of AroG lines #1, #2, #3 (*n* = 8–10) from EV (*n* = 7) 21 days after irrigation with 150 mM salt. EV is marked as 100%. Data are presented as the mean (±) SE. Significance (*p* ≤ 0.05) compared to EV is marked by asterisks.

To test tolerance to salt stress, 3-week-old propagated transgenic lines and EV that were grown on soil were supplemented with 150 mM NaCl. After 21 days, plant height, senescence level, plant vigor (see “Materials and Methods” section) and total dry biomass of the shoots were measured. The results showed that line #1 was more tolerant to 150 mM NaCl compared to EV since it accumulated 3.5-fold more biomass, had improved vigor (2-fold), and had less senescence phenotype (3-fold lower) compared to EV (Figure 7). Although lines #2 and #3 had a biomass similar to EV, line #2 showed significantly higher tolerance in the height and plant vigor parameters (1.7-fold higher in both) (Figure 7B).

The ability to cope with oxidative stress was also examined in the leaves. The transgenic lines showed high TPC, which could function as scavengers of reactive oxygen species (Das and Roychoudhury, 2014). Leaf discs were exposed to oxidative stress caused by methyl viologen. Bleaching content resulting from chlorophyll degradation was calculated and marked as% of green level compared to non-oxidative conditions. Leaf discs of line #1 had similar bleaching to EV discs in 1 μM methyl viologen (labeled pq), but when exposed to 2 μM and 5 μM methyl viologen, they were less bleached [95 and 90% green level of that detected in the control of non-treated plants (0 pq)], compared to EV (having 80% green level of its control) (Supplementary Figure S7). At 2 and 5 μM methyl viologen, line #2 had 84 and 87% green level of its control, and line #3 had 93 and 87% green level of its control, but these values were insignificant compared to the bleaching of EV at 2 and 5 μM methyl viologen (Supplementary Figure S7).

For drought stress experiments, we used plants of similar size and age. The plants were irrigated normally for 3 weeks (4–6 developed leaves), afterward the irrigation was stopped for 10 days. Parameters of senescence level and plant vigor score were measured (Supplementary Figure S8). The plants were then watered fully for 3 days to full recovery, and a second drought period commenced for 10 days. At the end of the second drought, parameters of height, dry biomass and relative water content (RWC) were measured (Supplementary Figure S8). The data showed that AroG lines did not exhibit improved tolerance to drought compared to EV, as lines #1 and #2 resembled EV in all of the parameters that were measured. Line #3 had an improved vigor score by 1.6-fold compared to EV and a 40% lower senescence level (Supplementary Figure S8).

### The Transgenic AroG Plants Were More Tolerant to the Parasitic Plant, *P. aegyptiaca*

It had been found previously that the application of higher levels of certain amino acids can inhibit the germination of the obligate parasite, *Orobanche ramosa* seeds (Vurro et al., 2006; Fernández-Aparicio et al., 2013, 2017). This suggests that high levels of amino acids can alter the parasite metabolism and cause growth inhibition. These findings encouraged us to determine whether the AroG lines would be more tolerant to *P. aegyptiaca*, a close relative of *O. ramosa*. *P. aegyptiaca* is an obligatory root parasite lacking chlorophyll that parasitizes many dicotyledonous crops, causing tremendous losses in crop yield and quality worldwide (Joel et al., 2007).

**FIGURE 8:**
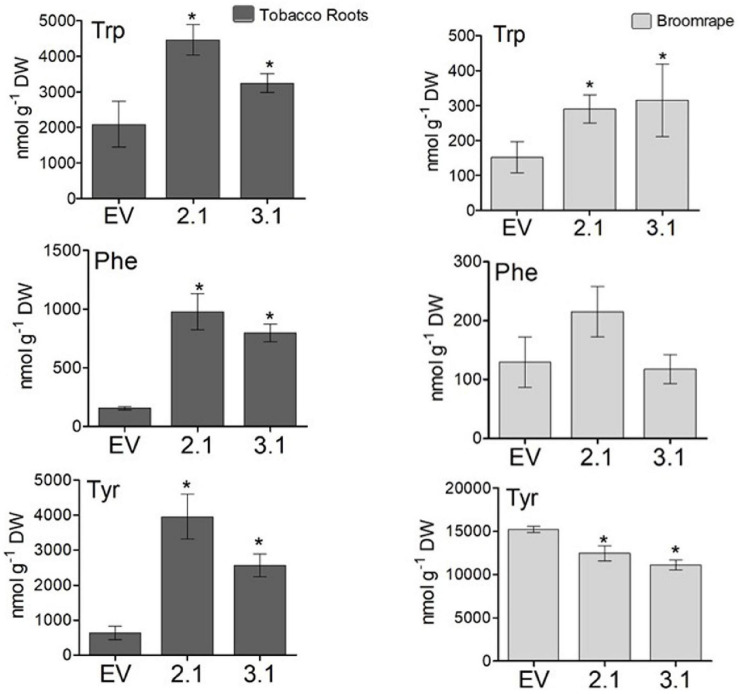
The levels of aromatic amino acids in the roots of the transgenic plants overexpressing the bacterial AroG gene and in *P. egyptiaca*. The levels of AAAs were detected by GC-MS analysis in the roots of AroG lines #2.1 and #3.1, in EV (dark gray) and in *P. aegyptiaca* (light gray). Data are presented as the mean ± SE of five different tobacco plants or of *P. aegyptiaca* that were collected from five different pots. Asterisks represent statistical significance (*p* ≤ 0.05) compared to the control (EV) was determined using the Student’s *t*-test.

**FIGURE 9:**
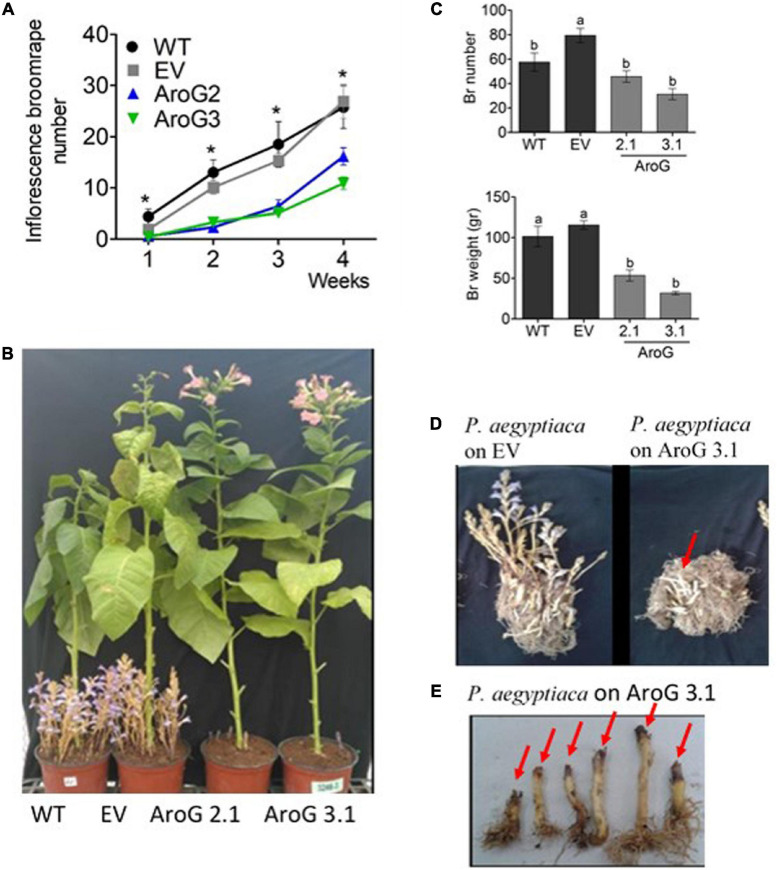
Phenotype of *Phelipanche aegyptiaca* that grew on homozygous AroG lines #2.1 and #3.1. **(A)** Inflorescence *P. aegyptiaca* number emerging from the soil per pot over 4 weeks. The count begins when the first *P. aegyptiaca* shoot emerged from the soil. Significant difference between WT/EV and AroG lines was determined using the Student’s *t*-test (*p* < 0.05) and is marked by asterisks. **(B)** Representative of photos showing the phenotype of *P. aegyptiaca* grown on AroG lines #2.1 and #3.1 and the control WT/EV at the end of the experiment (4 weeks from the time that *P. aegyptiaca* inflorescence started to emerge from the soil). **(C)** Weight and number of *P. aegyptiaca* per pot. Measurements were taken after the roots of the tobacco plants were washed from the soil and all of the *P. aegyptiaca* were collected. Different letters represent statistical significance (*p* ≤ 0.05) using the Tukey-Kramer test. Data are presented as the mean ± SE of *P. aegyptiaca* that were collected from 6 to 8 different tobacco plants for each set. **(D)** The phenotype represents tobacco roots and *P. aegyptiaca* developed on EV and on transgenic tobacco line #3.1. The red arrow marks *P. aegyptiaca* attached to the roots that did not emerge from the soil. **(E)** The phenotype represents inflorescence shoots of *P. aegyptiaca* grown on line #3.1. The red arrow marks the black edges of *P. aegyptiaca*.

To test the tolerance of the transgenic plants to *P. aegyptiaca* infection, homozygous plants of the second generation, offspring of line #2 (#2.1) and line #3 (#3.1) with normal phenotypes, were used. Although the levels of AAAs were not significantly altered in the parents of these lines compared to WT/EV (Figure 4), in the leaves of these homozygous lines, #2.1 and #3.1, the level of tyrosine significantly increased by 3.3- to 3.5-fold, and the level of phenylalanine significantly increased by 2- to 3.8-fold compared to EV (Supplementary Table S3). The level of tryptophan, however, did not significantly changed. Since *P. aegyptiaca* is a root parasite, we also measured the levels of these AAAs in the roots of lines #2.1, #3.1, to define that compared to the roots of EV, the levels of tryptophan increased by 1.5- to 2-fold, tyrosine by 4- to 6-fold, and phenylalanine by 5- to 6-fold, respectively (Figure 8). The levels of AAAs were similar in WT and EV plants (Supplementary Table S3). Four-week-old transgenic plants and their controls (WT/EV) were transferred to soil mixed with seeds of *P. aegyptiaca*. Twelve weeks later, *P. aegyptiaca* inflorescence started to emerge from the ground. The numbers of *P. aegyptiaca* inflorescence that emerged from the ground were counted every week over a period of 4 weeks. The results showed that at the first week of measurement, the numbers of parasites in the pots of lines #2.1 and #3.1 were 30 and 20% lower, respectively, compared to those detected in the pots of WT/EV (Figure 9A). At week 4, the numbers of parasites were 60 and 40% lower, respectively, compared to the WT/EV pots (Figure 9A). A difference was also detected in the developmental state of the parasite that had delayed development on the AroG lines. *P. aegyptiaca* that developed on AroG lines were at a stage of underground pre-emergent or post-emergent shoots with flower buds, while most of the *P. aegyptiaca* that developed on WT/EV were fully flowered (Figure 9B).

At the end of the experiment (4 weeks from the time that *P. aegyptiaca* inflorescence started to emerge from the soil), the roots of the tobacco plants were washed from the soil in order to count the total number of *P. aegyptiaca* that were attached to the roots, including those that remained underground. The results showed that the total *P. aegyptiaca* number in lines #2.1 and #3.1 pots was lower by 42–61%, respectively, compared to EV but did not differ from WT (Figure 9C). Due to the observation that the *P. aegyptiaca* grown on lines #2.1 and #3.1 were less developed than those grown on WT/EV, the biomass of total *P. aegyptiaca* was measured. The biomass was lower by 54–73% in pots of lines #2.1 and #3.1, respectively, compared to the control (Figures 9C,D). Notably, some of the edges of the parasites’ inflorescences attached to the roots of these AroG lines exhibited a brown/black color and looked damaged, and they were unable to develop further (Figure 9E). These data demonstrate that *P. aegyptiaca* growth was significantly inhibited when it was attached to AroG plants.

To show indications of reasons for the inhibition effect, the shoots of *P. aegyptiaca* were analyzed for the levels of AAAs by GC-MS. The level of tryptophan was significantly higher by 1.9- and 2-fold in *P. aegyptiaca* that were grown on lines #2.1 and #3.1, respectively, compared to those grown on EV. However, the level of phenylalanine did not differ significantly between these lines, and the level of tyrosine decreased slightly but significantly by 19 and 26% in *P. aegyptiaca* grown on #2.1 and #3.1 plants, respectively (Figure 8). The levels of AAAs were similar in *P. aegyptiaca* grown on WT and EV (Supplementary Table S3).

**FIGURE 10:**
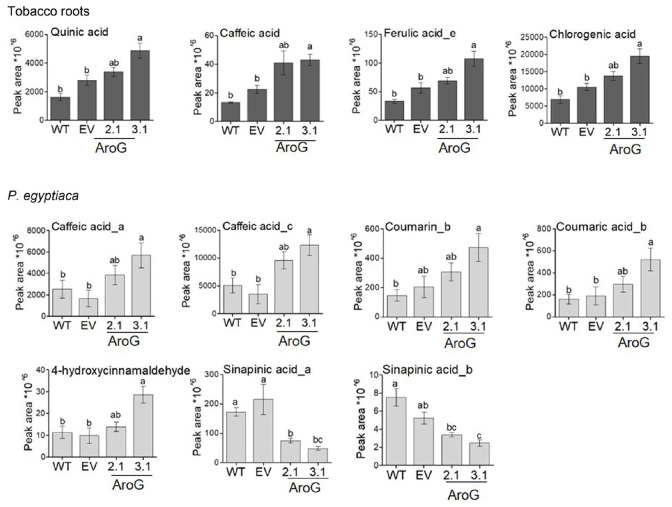
Levels of phenylpropanoids as detected by LC–MS/MS analysis in roots and in the parasite. Levels of the compounds in transgenic AroG lines #2.1 and 3.1, EV and WT (dark gray) and *Phelipanche aegyptiaca* attached to the roots of these plants (light gray). Data are presented as the mean ± SE of six different plants. Different letters represent statistical significance (*p* ≤ 0.05), which was determined using the Tukey-Kramer test. The figure shows only the phenylpropanoids that changed significantly.

The AAAs can be used in *P. aegyptiaca* to produce phenols and other metabolites that might accumulate and thus inhibit the growth of the parasite. Hydroxycinnamic acid compounds that belong to the phenylpropanoids may affect growth retardation, since feeding analysis with these compounds showed that they can reduce the radicle growth of *O. crenata* (Fernández-Aparicio et al., 2013). Therefore, we measured the levels of several phenylpropanoids in the tobacco roots and in *P. aegyptiaca* plants using LC-MS/MS. The results showed that the levels of several phenylpropanoids contents were changed. Compared to EV roots, the roots of line #3.1 had a significant increase in the levels of caffeic acid (1.9-fold), quinic acid (1.7-fold), chlorogenic acid (1.9-fold) and ferulic acid isomer (1.9-fold). The levels of these compounds also increased in the roots of line #2.1, although the elevation did not differ significantly from WT/EV (Figure 10 and Supplementary Table S4). We assumed that the high levels of AAAs and phenylpropanoids found in the #3.1 roots could affect the levels of phenylpropanoids in *P. aegyptiaca* grown on this line. To test this assumption, this analysis was also performed on the parasite shoots. *P. aegyptiaca* grown on line #3.1 showed significantly higher levels of two isomers of caffeic acid (3.4- and 4.1-fold), 4-hydroxycinnamaldehyde (2.9-fold), coumarin (2.3-fold), and comaric acid (2.7-fold) compared to those grown on EV (Figure 10 and Supplementary Table S4). *P. aegyptiaca* that were grown on line #2.1 showed a similar trend, although the elevation was not significant compared to those grown on WT/EV (Figure 10 and Supplementary Table S4). Unlike these elevations, the level of two isomers of sinapinic acid decreased significantly by 65 and 77% for isomer a, respectively, and 35 and 53% for isomer b in *P. aegyptiaca* that were grown on transgenic lines #2.1 and #3.1, respectively, compared to the EV control (Figure 10). Although these changes in phenylpropanoids levels, the levels of total phenols content (TPC) inside the parasite was not significantly altered in *P. aegyptiaca* that were grown on AroG lines and those grown on WT/EV (Supplementary Figure S9).

**FIGURE 11:**
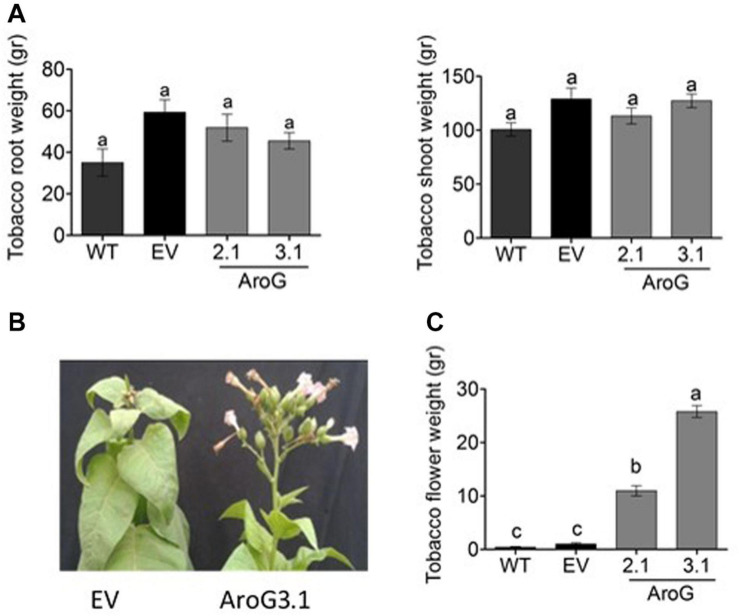
Phenotype of homozygotes AroG lines # 2.1 and #3.1 and WT/EV that were infected by *Phelipanche aegyptiaca*. **(A)** Weights of shoots and roots of the tobacco plants. **(B)** Represents the edge of the shoots and inflorescence of the tobacco EV and AroG line #3.1 plants. **(C)** Weight of tobacco flowers. Data are presented as the mean ± SE of total flowers that were collected from 6 to 8 plants for each set. Different letters represent statistical significance (*p* ≤ 0.05), which was determined using the Tukey-Kramer test.

The normal development of the host plants is usually impaired when they are infected by *P. aegyptiaca*. Therefore, we measured the weights of the tobacco plants to find out that the shoots and roots weights of lines #2.1 and #3.1 were similar to WT/EV (Figure 11A). However, the infected WT/EV plants developed a small number of abnormal flowers (Figure 11B), unlike lines #2.1 and #3.1 that developed normal inflorescences and flowers (Figures 10B, 11B). Accordingly, total flower weight was 10- to 25-fold higher, at these transgenic lines compared to WT/EV (Figure 11C). This phenotype, together with the lower levels of inflorescence shoots of the parasite that emerged from the ground, suggest that the transgenic lines are more tolerant to the parasite than the WT/EV control plants.

## Discussion

### The Effect of High Expression Level of AroG on the Phenotype of Tobacco Plants

Unlike transgenic *A. thaliana* and petunia plants overexpressing the bacterial AroG gene, which showed a similar morphological phenotype to their control plants (Tzin et al., 2012; Oliva et al., 2015), about 30% of the transgenic tobacco plants showed a severely abnormal phenotype (Figure 1). All of the plants with an abnormal phenotype (lines #4–6) had high protein expression levels of AroG, while plants with a normal phenotype were divided into two sub-set: those with low expression of AroG protein (e.g., lines #2–3); and those with high expression (e.g., line #1). The reason for the abnormal phenotype is still unknown, but since the level of tryptophan, the precursor for auxin synthesis, significantly increased in transgenic lines #4–6 (Figure 4), we tried to determine if auxin is involved. No significant evidence was found for a higher level of auxin in AroG tobacco lines. Thus, we assumed that the abnormal phenotype in lines #4–6 results from a high accumulation of AAAs and their associated secondary metabolites (Figure 4). This assumption was supported by an analysis of transgenic *A. thaliana* plants expressing a mutated gene of arogenate dehydratase 2 (ADT2), the last enzyme in the phenylalanine biosynthesis pathway called *padt*2-1D. These plants had a 70-fold increase in phenylalanine, 67-fold increase in tryptophan and 9.8-fold increase in tyrosine levels compared to WT. In addition, the levels of salicylic acid and several metabolites related to phenylpropanoid increased significantly (Huang et al., 2010). The *padt*2-1D transgenic plants exhibited a relatively similar phenotype to transgenic tobacco lines #4–6, which included an altered rosette leaf morphology as the leaves were narrower with curled edges, and some plants in the segregating populations also had a dwarf phenotype and didn’t produce seeds (Huang et al., 2010). A similar morphological phenotype was also exhibited in *A. thaliana* plants that overexpressed ADT4 or ADT5, as they had a dwarf phenotype with narrow, small and curled leaves, and some plants were sterile (Chen et al., 2016).

### The Effect of High Expression Level of AroG on the Metabolic Profile of Tobacco Plants

The primary and secondary metabolites that were detected on tobacco leaves showed a positive correlation between AroG protein accumulation and metabolites that related to the shikimate pathway, AAAs, and their derived metabolites (Figure 4). Similar results were obtained when AroG was overexpressed in other plants and tissues (*A. thaliana*, petunia, tomato fruit and grape cell culture; Tzin et al., 2012, 2013; Manela et al., 2015; Oliva et al., 2015). As we had found for tobacco lines #1 and #4–6, the levels of all three AAAs increased significantly in the leaves of transgenic AroG petunia plants compared to control plants (Table 1; Oliva et al., 2015). Expressing this heterologous gene in *A. thaliana* and red tomato fruits resulted in significantly increased levels of phenylalanine and tryptophan compared to control, while tyrosine was not altered significantly (Table 1; Tzin et al., 2012, 2013). In the grape cell culture, only phenylalanine and tyrosine increased (Table 1; Manela et al., 2015). These data show that the expression of AroG mostly affected the level of phenylalanine. In addition to the accumulation of AAAs, the increased levels of shikimate in tobacco, *A. thaliana* and ripe red tomato fruits that expressed AroG (Table 1) indicate that DAHPS serves as a major regulator of flux throughout the shikimate pathway.

**TABLE 1:** The effect of AroG expression on the levels of shikimate pathway metabolites and AAAs secondary metabolites in different transgenic plants.

	Shikimate	Phenylalanine	Tryptophan	Tyrosine	Shikimate metabolites and AAAs derivatives secondary metabolites	
Tobacco leaves	> 7	>43	> 10	>24	Quinate, Phenylpyruvate, 4-hydroxybenzoate, Phenyllactate and Phenylethylamine	
*A. thaliana* Se*edli*ng	> 30	>180	> 2.6	NS	Prephenate, Phenylacetonitrile, homogentisate, 4-Hydroxybenzoate, Coumarate hexose derivatives, Ferulate hexose derivatives, Ferulic acid derivatives, Sinapoyl hexose derivative, sinapate, Sinapyl alcohol, Coniferin, 2-phenylethyl glucosinolate, 4-Methoxyindole glucosinolate	Tzin et al., 2012
Petunia leaves	NR	> 122	>8	>14	Phenyllactate, Prephenate, 5 trans Caffeoyl quinate, 4 Caffeoyl Quinate, rosmarinate, Salysilate Glucopyranoside, Sinapate, trans- Caffeate, 1 Benzylglucopyranoside, 3,4-Hydroxyphenyllactate, Tocopherol, Pyrogallol, Dihydroxyphenylalanine, Ferulate, Hydroquinone	Oliva et al., 2015
Tomato fruits Ripe red	> 62	> 88	> 4	>171	3-Caffeoylquinic acid, 4-Caffeoylquinic acid, Coumaric acid, Coumaric acid hexoside or derivative, Kaempferol-glucose-rhamnose, Naringenin chalcone, Tricaffeoylquinic acid	Tzin et al., 2013
Grape cell culture	> 3.5	>40	ND	> 800	Phenylpyruvate, 3-hydroxy phenylacetate, Hydroquinone, 4-Hydroxyphenyl b-glucopiranoside, Coumarate, Resveratrol, 4-Hydroxy-benzoate, Dihydroquerccetin, Epicatechin	Manela et al., 2015

*Data are given in fold change above the control. NS, not significant; NR, not reported.*

The high levels of AAAs found in AroG tobacco plants affected the levels of their downstream secondary metabolites since the levels of the phenylalanine-derived metabolites, phenyllactate, 4-hydroxybenzoate and phenylethylamine, accumulated in AroG plants compared to the control (Figure 4). This is in accordance with other transgenic plants and tissues overexpressing the AroG gene, although in each plant species or tissue type, different metabolites that are driven from the AAAs were altered (Table 1). Taken together, the data suggest that the overexpression of the bacterial AroG gene significantly enhanced flux toward the shikimate pathway, resulting in higher levels of AAAs and their associated secondary metabolites, which change depending on the different regulatory points and the biosynthesis genes found in these plants.

The primary metabolic profile of tobacco plants showed that although transgenic lines #1 and #4–6 had a significant increase in AAAs, only mild changes were observed in the contents of other amino acids (Figure 6 and Supplementary Table S1). Most of the soluble amino acids did not change significantly compared to WT/EV, with the exception of serine, which decreased, most probably since it is used for the tryptophan biosynthetic pathway as a nitrogen source (Tzin and Galili, 2010), and methionine, a member of the aspartate family amino acids, which decreased in lines #4–6, indicating a possible interaction between the aspartate family and AAA metabolic networks. The mild effect of increased AAAs content on other amino acids was also detected in *A. thaliana* and petunia, which overexpressed AroG. In *A. thaliana* seedlings, only alanine increased significantly (Tzin et al., 2012), while in petunia leaves, the levels of glutamine and asparagine decreased (Oliva et al., 2015). These two amino acids are nitrogen donors in the final step of keto-acid conversion into phenylalanine and tyrosine (Oliva et al., 2015). Mild changes in the levels of amino acids were also detected in transgenic *padt2-1D A. thaliana* lines, in which the increased levels of AAAs were accompanied by elevated levels of arginine, valine, and leucine (Huang et al., 2010).

The expression of AroG had a more significant effect on the sugars profile. In tobacco lines that highly expressed AroG, there was an increase in the levels of several sugars such as fructose, glucose, trehalose, melibiose, β-D-galactopyranoside, and gentabiose (Figure 6 and Supplementary Table S1). All of the above-mentioned sugars and additional sugars increased in the leaves of two petunia lines that overexpressed AroG (Oliva et al., 2015). Unlike tobacco and petunia, transgenic *A. thaliana* seedlings showed no significant changes in their sugars levels (Tzin et al., 2012).

Overall, the data of the primary metabolic profile indicate that enhanced shikimate pathway activity in tobacco plants mostly increase the carbon metabolites (e.g., sugar, TCA cycle and shikimate) and less the nitrogen metabolites (e.g., amino acid and polyamines). Based on the results obtained from other transgenic plants, the effect of AroG on the metabolic profiling varies between different genotypes.

### Contribution of High AAA Levels to Abiotic Stress Tolerance

Plants, in response to abiotic stresses (such as salt, drought or oxidative), induce the shikimate pathway and its downstream metabolites (AAAs and phenylpropanoids) to gain better adaptability to the stress (Dixon and Paiva, 1995; Pandey et al., 2015; Francini et al., 2019; Sharma et al., 2019). An example of the effect of a high level of AAAs in stress response comes from a recent study showing that the direct or indirect application of phenylalanine can increase tolerance to the fungal pathogen *Botrytis cinerea* in tomato and petunia leaves by enhancing the levels of anti-fungal phenylalanine-derived metabolites (Oliva et al., 2019). Also, when tryptophan was added to the growth medium of *A. thaliana* seedlings, it was revealed that they were more tolerant to stress caused by cadmium (Cd), a phytotoxic metal in soils (Sanjaya et al., 2008). In accordance, tryptophan-overproducing *trp5-1* plants were more tolerant to Cd while tryptophan auxotroph *trp2-1* was more sensitive to Cd (Sanjaya et al., 2008).

To determine if AroG tobacco plants would be more tolerant to abiotic stress, we tested lines #1–3 for their ability to cope with salt, oxidative and drought stresses. Line #1, which accumulated a high level of AroG protein and high contents of AAAs and their derivatives, showed improved salt tolerance in all tested parameters, line #2 exhibited higher levels of only two parameters, and line #3 did not show an advantage over the control WT/EV plants (Figure 7). The higher ability of AroG line #1 might be related to the observation that it had slightly higher levels of proline and sugars (Figure 6), which are known to function as osmolytes (Bolouri-Moghaddam et al., 2010; Verslues and Sharma, 2010). *A. thaliana* transgenic plants that overexpressed the mutant gene of *adt2* and accumulated high levels of phenylalanine also exhibited enhanced tolerance to salt stress as detected by its root length (Huang et al., 2010), suggesting that higher levels of phenylalanine contributed in some manner to salt tolerance. An efficient response in a plant to salt stress demands a multifactor response (Francini et al., 2019), therefore, it could be that changes in several metabolites that associated to the phenylpropanoids together contributed to better performance of AroG line #1 under stress.

In response to oxidative stress, the data indicate that AroG plants could have a minor advantage over WT/EV. This could be the result of increased TPC levels found in AroG plants, since phenols have ROS scavenging activity (Das and Roychoudhury, 2014). The response to drought stress did not show a significant advantage of AroG lines over the control plants, suggesting that the metabolic changes in tobacco AroG are less effective in adaptation to this stress.

### The Transgenic Plants Were More Tolerant to Infection Caused by *P. aegyptiaca*

Infection of AroG plants with *P. aegyptiaca* showed that plants with a high level of AAAs can inhibit the development of the parasitic plant. The inhibitory activity of certain amino acids on the germination and growth of broomrape (*P. ramose*, *Orobanche crenata*, and *Orobanche minor*) has been previously studied by adding certain amino acids to the growth media (Vurro et al., 2006; Fernández-Aparicio et al., 2013, 2017). For example, the application of exogenous methionine to the soil caused a strong reduction in the number of emerged *P. aegyptiaca* shoots, as well as in their dry and fresh weights, without causing a significant effect to the tomato plants host (Vurro et al., 2006). Similarly, when applied to tomato roots, methionine strongly reduced the number of developed tubercles of *P. ramosa* (Vurro et al., 2006). The inhibitory effect on germination and radicle growth was also found when each of the AAAs were added to the growth medium of *O. minor* (Fernández-Aparicio et al., 2017). Field experiments showed that irrigation with 20 mM tryptophan led to a 39% reduction in *O. minor* emergence above soil compared to the control (Fernández-Aparicio et al., 2017). Tryptophan added to the growth medium also inhibited *O. crenata* radical growth (Fernández-Aparicio et al., 2013). These findings suggest that certain amino acids applied exogenously can inhibit the growth of the parasitic plants. However, these previous studies used feeding experiments and not plants that have higher endogenous levels of amino acids.

In the current study, we demonstrated the potential of plants having a high level of AAAs to inhibit the growth of the parasitic plant *P. aegyptiaca*, without significantly affecting the phenotype of the host. The transgenic tobacco AroG lines were more tolerant to the parasitic plants, since, unlike EV and WT, the infected AroG lines had normal development and they had flowers. The growth of the parasitic plants was inhibited in the AroG lines as they had a significantly lower number of inflorescent shoots compared to WT/EV. Moreover, part of the *P. aegyptiaca* apical meristem turned black and their development ceased.

Although AroG roots accumulated higher levels of all three AAAs compared to WT/EV plants, the parasitic plants accumulated higher amounts only of tryptophan, while the level of phenylalanine remain unchanged and that of tyrosine decreased (Figure 9). Therefore, we assume that the inhibition of *P. aegyptiaca* development is due to changes in AAAs-derived metabolites. Support of this assumption comes from observations that *O. cumana* that grow on resistant sunflower plants accumulated higher phenolic compounds compared to *O. cumana* that grow on susceptible sunflower plants (Echevarría-Zomeno et al., 2006). Furthermore, the addition of scopoletin to the growth medium inhibits *O. crenata* seed germination and radicle growth and causes cell necrotic-like darkening in young radicles (Fernández-Aparicio et al., 2013). This metabolite is derived from the phenylpropanoid pathway and synthesized via cinnamates, and can accumulate in different plant species such as *A. thaliana* and cereal roots (Baghestani et al., 1999; Kai et al., 2008). The inhibitory effect of shikimate pathway-derived metabolites on plant growth was also seen when exogenous applications of three hydroxycinnamic acids, caffeic acid, p-coumaric acid and ferulic acid, induced a reduction in *O. crenata* radicle growth (Fernández-Aparicio et al., 2013). The roots of lines #3.1, which exhibit the highest tolerance to *P. aegyptiaca*, had significant higher levels of caffeic acid, ferulic acid, quinic acid and chlorogenic acid compared to WT/EV roots, indicating that the accumulation of these metabolites in the roots of the host plants can affect the growth of the parasitic plants. In addition, caffeic acid, coumarin, coumaric acid and 4-hydroxycinnamaldehyde were elevated in *P. aegyptiaca*, which was attached to the roots of line #3.1. This suggests that these compounds can be involved in *P. aegyptiaca* growth inhibition. Some of these metabolites can cause growth inhibition in different plant species, as reported previously for caffeic acid, coumaric acid and cinnamic acid derivatives that were applied exogenously (Zanardo et al., 2009; Orcaray et al., 2011; Fernández-Aparicio et al., 2013).

The increased levels of hydroxycinnamic acid were accompanied by a significant reduction in the levels of sinapinic acid (also called sinapic acid) in *P. aegyptiaca* that grow on lines #3.1 and #2.1. The relationship between accumulations of hydroxycinnamic acid, p-coumaric acid, caffeic acid and ferulic acid in the roots and lower levels of sinapinic acid was previously detected in pea plants that were treated with herbicides chlorsulfuron and imazethapyr, which inhibit acetolactate synthase (ALS) (Orcaray et al., 2011).

Taken together, the results show that higher expression levels of AroG in the host plant (tobacco) can inhibit the growth of the parasitic plant *P. aegyptiaca* and bring a novel potential way to manage such parasitic weeds.

## Conclusion

This study aimed at gaining more knowledge about the effect of increasing the AroG gene encoded to feedback-insensitive 3-deoxy-D-arabino-heptulosonate 7-phosphate synthase (DAHPS), the first enzyme of the shikimate pathway, on the accumulation of AAAs and their associated metabolites in tobacco plants. We also aimed at revealing the effect on the primary metabolic profile, tolerance to abiotic stress, and the ability to cope with *P. aegyptiaca*, a parasite plant. Our study is in agreement with previous studies, indicating that DAHPS serves as a major regulator of flux throughout the shikimate pathway and that most of the carbon flux goes toward phenylalanine, whose level increased up to 43-fold. The significant increase in phenylalanine resulted in increased levels of metabolites that belong to the phenylpropanoid pathway, including 4-hydroxybenzoate, phenyllactate and phenylethylamine. These changes were accompanied by mild changes in the plants’ primary metabolites, most of which were in sugars content. In addition, our results provide evidence that plants with high levels of AAAs and their related metabolites have improved tolerance to salt stress and the potential to inhibit the development of the parasitic plant, *P. aegyptiaca*.

## Data Availability Statement

The datasets presented in this study can be found in online repositories. The names of the repository/repositories can be found in the article/Supplementary Material.

## Author Contributions

YH, MO, and RA: experimental design and manuscript preparation. AG, ED, and MO: conducting the experiments. AG, MO, RS, and YH: data analysis. All authors have read and approved the manuscript.

## Conflict of Interest

The authors declare that the research was conducted in the absence of any commercial or financial relationships that could be construed as a potential conflict of interest.

## Footnotes

1 www.nist.gov

2 https://imagej.nih.gov/ij/

3 http://metaboanalyst.ca/

4 http://www.graphpad.com/

## Supplementary Material

Supplementary Figure 1 Schematic diagram of the aromatic amino acid (AAAs) biosynthesis pathway in plants. The main related metabolites from each of the AAAs are indicated in the blue framed box. Only regulatory enzymes are mentioned. DAHPS, 3-deoxy-d-arabino-2-heptulosonate 7-phosphate synthase; CM, chorismate mutase; ADT, arogenate dehydratase; AS, anthranilatesynthate; ADH, arogenate dehydrogenase.

Supplementary Figure 2 Schematic diagram of the constructs used in the current study. Upper panel: the construct used to express the AroG gene. Lower panel: an empty vector (EV) used as a control. 35S Omega, cauliflower mosaic virus 35S promoter fused to the omega translation enhancer; TP, RuBisCO small subunit-3A plastid transit peptide; AroG, the bacterial feedback-insensitive AroG encoded to 3-deoxy-d-arabino-2-heptulosonate 7-phosphate synthase with a mutation at amino acid number 175; 3HA, three copies of the hemagglutinin epitope tag; OCS ter, octopine synthase terminator; LB, left border; RB, right border.

Supplementary Figure 3 Screening of the protein expression level of DAHPS in the T_0_ tobacco transgenic plants. The upper panel shows the immunoblot analysis made for the leaves of 12-week-old transgenic plants using antibodies against the 3HA epitope-tag that was fused to the AroG gene. The size of the DAHPS is 42 kDa. The lower panel shows Coomassie-blue staining of the protein profiles of tobacco leaves. Proteins (20 μg) extracted from the leaves were fractionated by SDS-PAGE. M, marker size; EV, empty vector. Each of these gels was run separately. Lines #1–3 (in the text) are plants No. 3, 8, and 10, respectively, and lines #4–6 are plants No. 16, 17, and 26. Plants that had abnormal shoot phenotype are marked by antistrike.

Supplementary Figure 4 Expression levels of two auxin response genes at WT, EV, and AroG transgenic tobacco lines as detected by qRT-PCR. Data are given as relative to protein phosphatase 2A subunit (PP2A) expression. Data are presented as the mean ± SE of three different biological repetitions. Asterisks represent statistical significance (*p* ≤ 0.05) of each AroG line from EV plants (using the Student’s *t*-test).

Supplementary Figure 5 Effect of auxin treatment on plant phenotype. **(A)** The phenotype of WT plants grown for 8 weeks on Nitsch medium supplemented with aphthaleneacetic acid (NAA) or phenylacetic acid (PAA). **(B)** The phenotype of 3-week-old tobacco WT plants sprayed with increasing concentrations (0.1, 1, 10, 100 μM) of indole-3-butyric acid (IBA), indole-3-acetic acid (IAA), indole-3-propionic acid (IPA), or tryptophan (TRP) for 10 days. Spraying with double-distilled water (DDW) was used as a control.

Supplementary Figure 6 Phenolic compounds found in leaves of AroG lines #1–6 and in EV/WT plants as measured at a wavelength of 280 nm using HPLC-DAD. Data are presented as the mean ± SE of four biological repetitions. Different letters represent statistical significance (*p* ≤ 0.05), which was determined using the Tukey-Kramer test.

Supplementary Figure 7 The response of AroG lines to oxidative stress. Eight mm discs (*n* = 8–12) of transgenic AroG #1, #2, #3, and EV leaves (*n* = 7) were exposed to different levels of in 0, 1, 2,5 μM methyl viologen (pq) for 20 h under 80–110 μE light conditions, and bleaching level was measured by ImageJ. Data are presented as a change (in%) from green level under the non-stress condition (0 pq) that was marked as 100%. Significance (*p* ≤ 0.05) from EV at the same condition is marked by asterisks.

Supplementary Figure 8 The response of AroG lines to drought stress. The plants were grown in a greenhouse as described in “Materials and Methods” section. Parameters of senescence level and plant vigor were measured after the first drought period of 10 days, and parameters of height, dry biomass and relative water content (RWC) were measured at the end of the second drought period of 10 days. Data are presented as the mean ± SE of six biological replicates. Significance (*p* ≤ 0.05) of each line compared to EV under the same conditions is marked by asterisks.

Supplementary Figure 9 Total polyphenol content (represented as g gallic acid equivalents per liter of water extract) in *P. aegyptiaca* grown on WT, EV, and AroG lines. Data are presented as the mean ± SE of *P. aegyptiaca* that were collected from five different pots. Different letters represent statistical significance (*p* ≤ 0.05), which was determined using the Tukey-Kramer test.

Supplementary Table 1 GCMS-metabolite dataset as detected in WT and transgenic plants.

Supplementary Table 2 List of primers used in the qRT-PCR analyses.

Supplementary Table 3 **(A)** The levels of aromatic amino acids in homozygous lines #2.1, #3.1, WT, EV and in P. aegyptiaca. **(B)** The levels of aromatic amino acid in the roots of WT and EV and in *P. aegyptiaca.*

Supplementary Table 4 Levels of phenylpropanoids detected by LC-MS/MS in *Phelipanche aegyptiaca (P. aegyptiaca)* and the roots of WT and transgenic plants.

Supplementary Text 4 Feeding experiments with auxin suggest that the phenotype of lines #4–6 is not related to high auxin levels.

Supplementary Text 6 Profile of phenolic compounds found in the leaves of AroG lines and WT/EV.
