# Supplementary material for: Enhanced Production of Aromatic Amino Acids in Tobacco Plants Leads to Increased Phenylpropanoid Metabolites and Tolerance to Stresses
Source: Front Plant Sci. 2021 Jan 12;11:604349. doi: 10.3389/fpls.2020.604349 (PMC7835393; doi:10.3389/fpls.2020.604349)
Supplement: Supplementary Table 1 — GCMS-metabolite dataset as detected in WT and transgenic plants. [file Data_Sheet_2.docx]

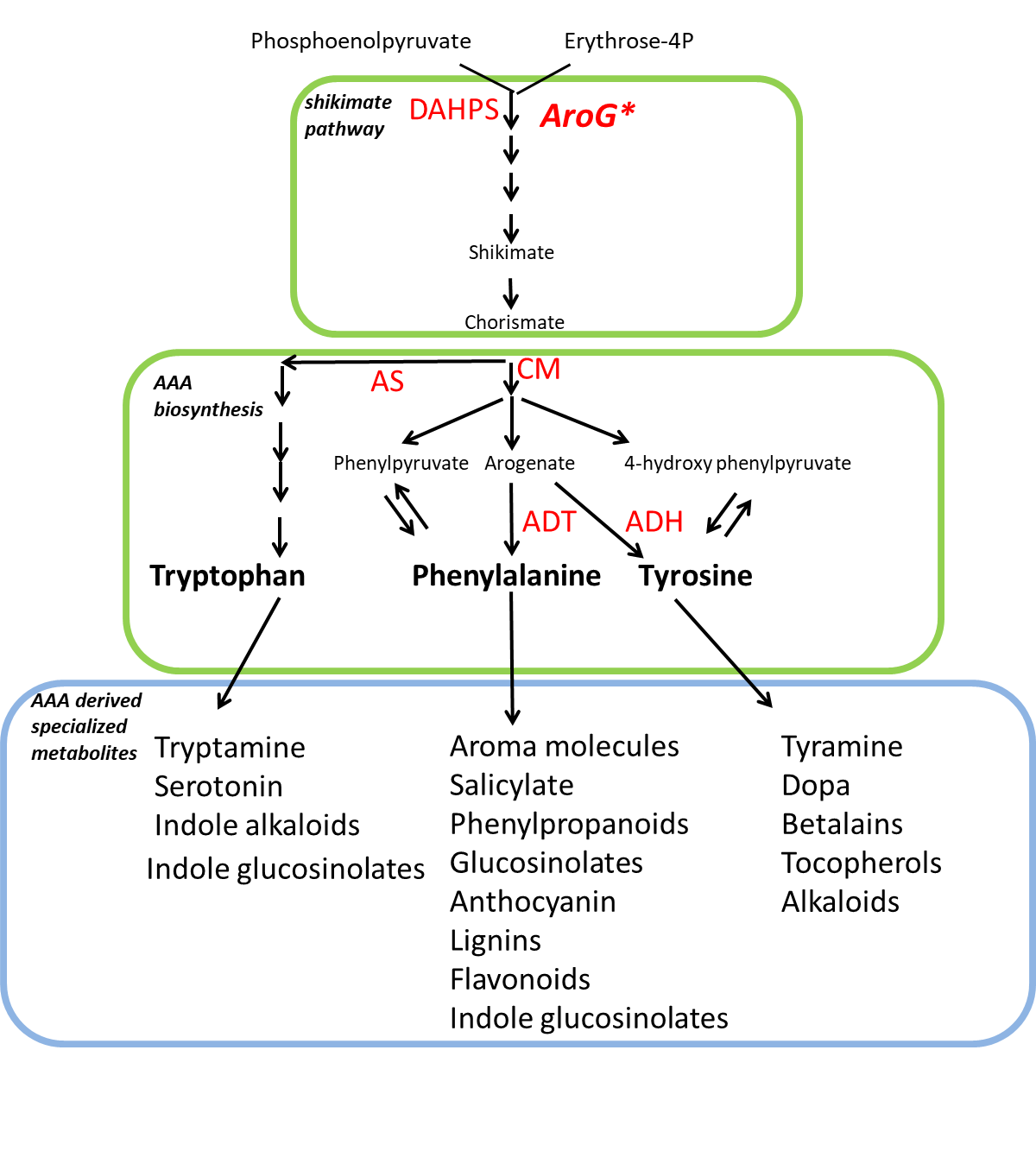


**FIGURE S1**: **Schematic diagram of the aromatic amino acid (AAAs) biosynthesis pathway in plants.** The main related metabolites from each of the AAAs are indicated in the blue framed box. Only regulatory enzymes are mentioned. Abbreviation: DAHPS, 3-deoxy-d-arabino-2-heptulosonate 7-phosphate synthase; CM, chorismate mutase; ADT, arogenate dehydratase; AS, anthranilatesynthate; ADH, arogenate dehydrogenase.

**EV**

**AroG**


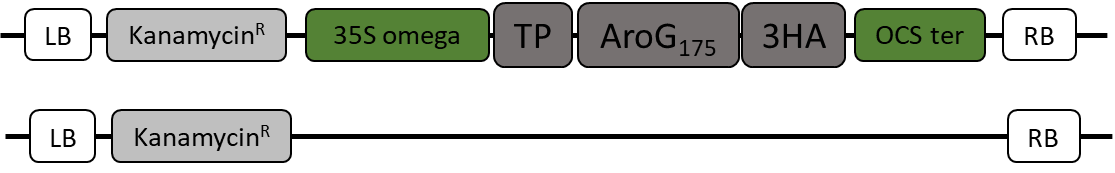


**FIGURE S2:** **Schematic diagram of the constructs used in the current study.** Upper panel: the construct used to express the AroG gene. Lower panel: an empty vector (EV) used as a control. Abbreviations: 35S Omega, cauliflower mosaic virus 35S promoter fused to the omega translation enhancer; TP, RuBisCO small subunit-3A plastid transit peptide; AroG, the bacterial feedback-insensitive AroG encoded to 3-deoxy-d-arabino-2-heptulosonate 7-phosphate synthase with a mutation at amino acid number 175; 3HA, three copies of the hemagglutinin epitope tag; OCS ter, octopine synthase terminator; LB, left border; RB, right border.

**M**


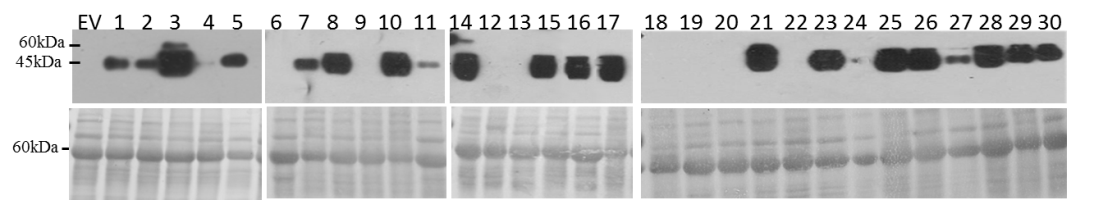


*

*

*

*

*

*

*

**FIGURE S3: Screening of the protein expression level of DAHPS in the** T_0_ **tobacco transgenic plants.** The upper panel shows the immunoblot analysis made for the leaves of 12-week-old transgenic plants using antibodies against the 3HA epitope-tag that was fused to the AroG gene. The size of the DAHPS is 42 kDa. The lower panel shows Coomassie-blue staining of the protein profiles of tobacco leaves. Proteins (20 µg) extracted from the leaves were fractionated by SDS-PAGE. M, marker size; EV, empty vector. Each of these gels was run separately. Lines #1-3 (in the text) are plants No. 3, 8 and 10, respectively, and lines #4-6 are plants No. 16, 17 and 26. Plants that had abnormal shoot phenotype are marked by antistrike.

**Text S4** **feeding experiments with auxin suggest that the phenotype of lines #4-6 is not related to high auxin levels.** The severe abnormal phenotype of lines #4-6 might be related to increased auxin levels that are produced from tryptophan. To gain an indication of this possibility, the transcript expression levels of the auxin response genes ARF8 and IAA4.3 were measured. These two genes are used as a marker of a high auxin level (Zhu et al., 2013, Dargeviciute et al., 1998). However, the expression levels of these genes did not differ significantly between the plants examined, with only one exception in line 5, which shows high expression of IAA4.3 (Supplementary Figure S3). To study further the effect of auxin on the phenotype of tobacco plants, WT plants were grown for eight weeks on Nitsch medium (Duchefa) containing synthetic auxin, aphthaleneacetic acid (NAA) or phenylacetic acid (PAA) at different concentrations. PAA is synthesized from phenylalanine and its metabolism and signaling effect appear to be similar to that of indole-3-acetic acid (IAA) (Sugawara et al., 2015). The addition of these compounds resulted in growth inhibition, but the phenotype was not similar to transgenic lines #4-6 (Supplementary Figure S4A, Figure 1A). In addition, different concentrations of indole-3-butyric acid (IBA), IAA, indole-3-propionic acid (IPA) and tryptophan were added for 10 days to WT plants grown in soil. Again, the phenotypes of these plants were not similar to transgenic plants lines #4-6 (Supplementary Figure S4B, Figure 1A). The results of these experiments did not provide evidence that the abnormal phenotype resulted from a higher auxin content.


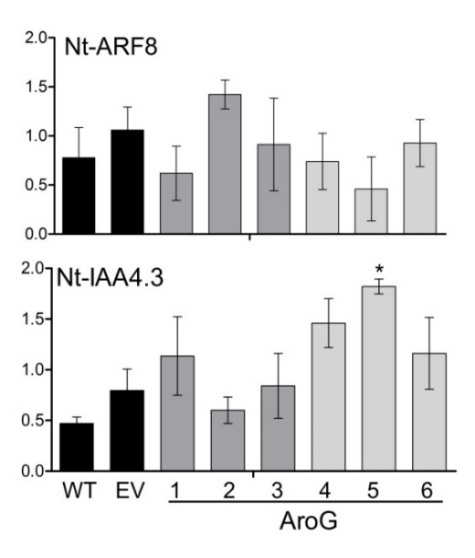


Relative expression levels

AroG

**FIGURE S4: Expression levels of two auxin response genes at WT, EV and AroG transgenic tobacco lines as detected by qRT-PCR.** Data are given as relative to protein phosphatase 2A subunit (PP2A) expression. Data are presented as the mean ± SE of three different biological repetitions. Asterisks represent statistical significance (p≤0.05) of each AroG line from EV plants (using the student t-test).


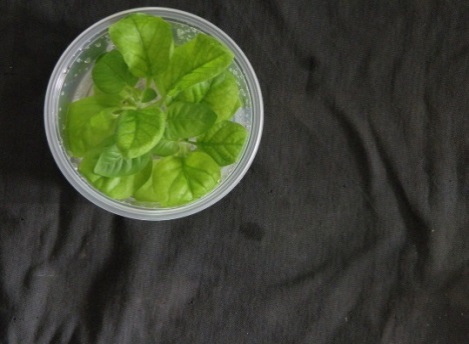

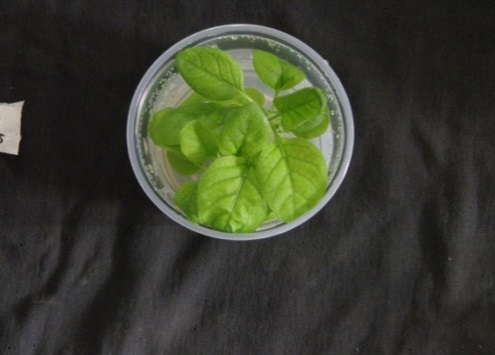

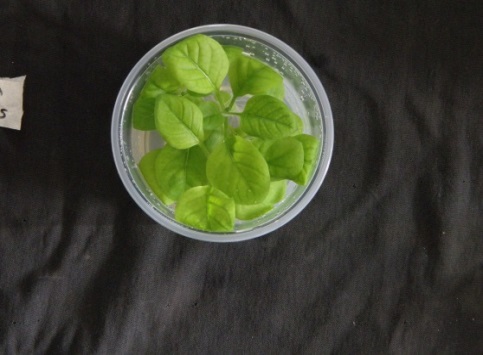

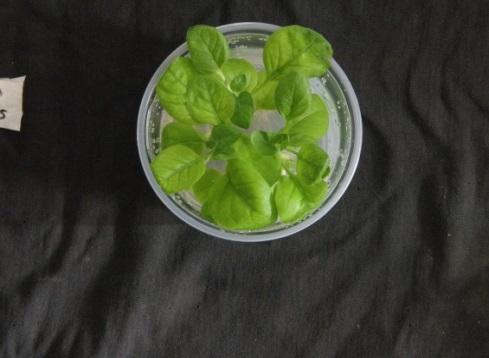

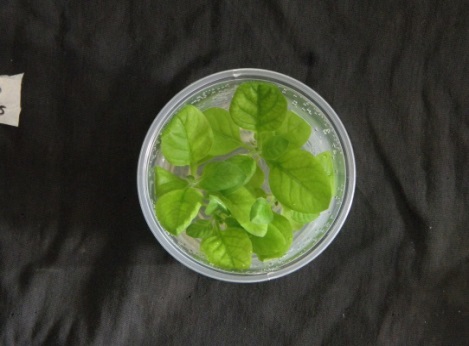

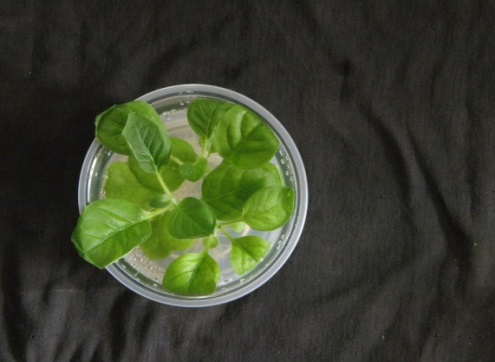

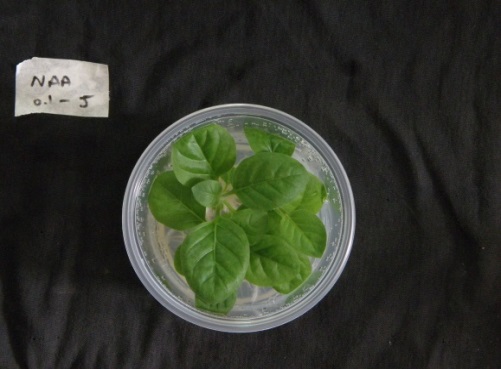

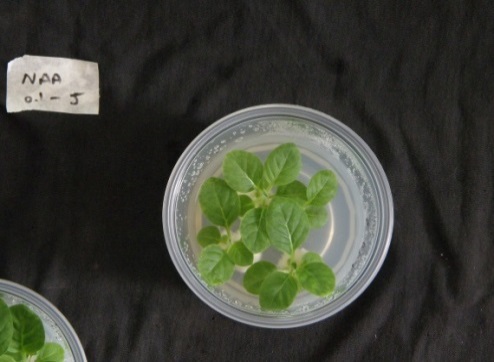

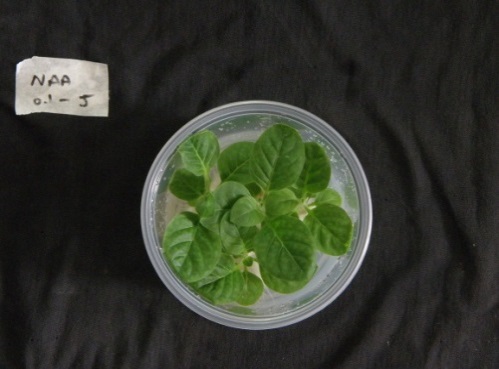


0.1 0.5 1 5 μM

NAA

PAA

0

**A**


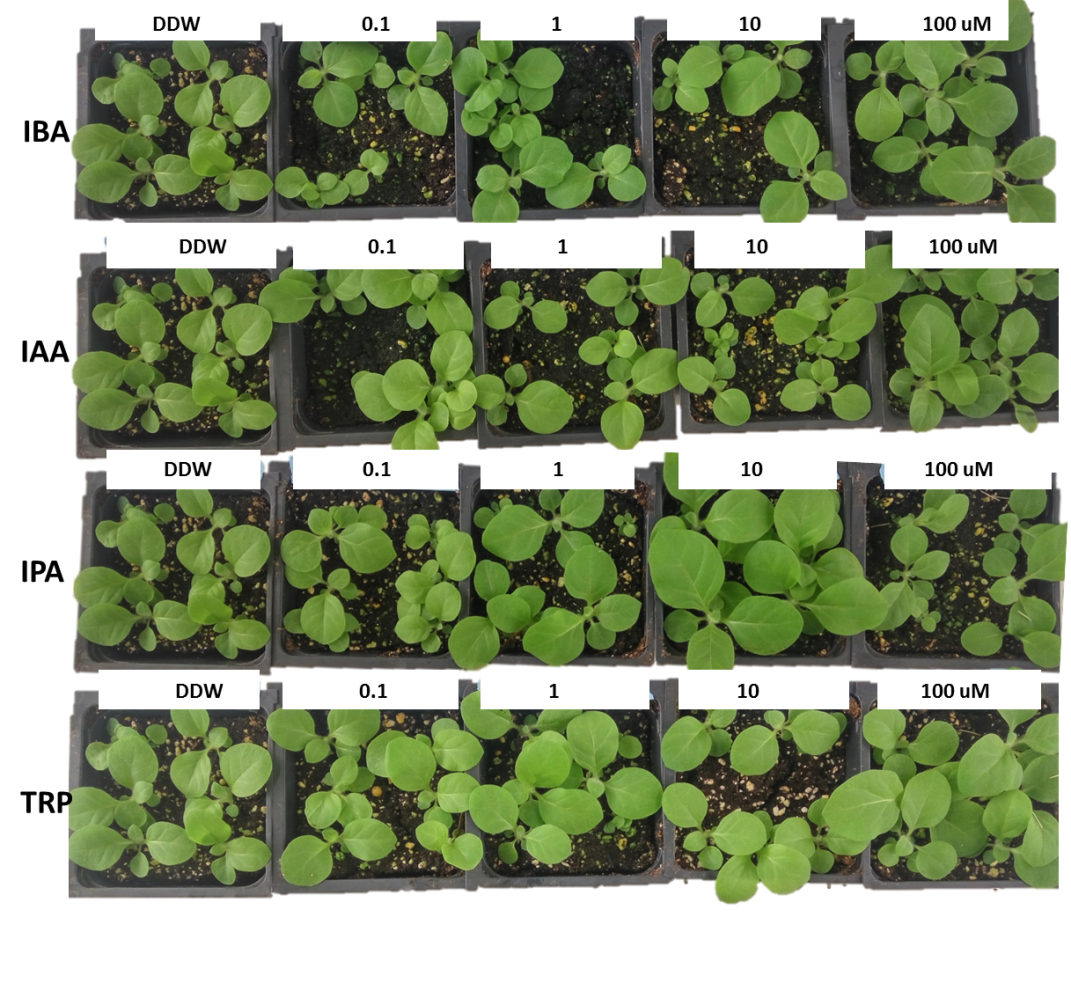


**B**

**FIGURE S5**: **Effect of auxin treatment on plant phenotype. (A)** The phenotype of WT plants grown for eight weeks on Nitsch medium supplemented with aphthaleneacetic acid (NAA) or phenylacetic acid (PAA). **(B)** The phenotype of three-week-old tobacco WT plants sprayed with increasing concentrations (0.1, 1, 10, 100 µM) of indole-3-butyric acid (IBA), indole-3-acetic acid (IAA), indole-3-propionic acid (IPA), or tryptophan (TRP) for 10 days. Spraying with double-distilled water (DDW) was used as a control.

**Text S6. Profile of phenolic compounds found in the leaves of AroG lines and WT/EV.** To test the differences in the profile of phenols in the leaves of AroG lines and WT/EV control, we used an HPLC-DAD analysis. Eight compounds were detected based on their retention times at a 280 nm wavelength; these phenols were not annotated. Compared to WT/EV, all of the AroG lines had higher levels of phenol no. 7 identified at a retention time (RT) of 18.1 min. The levels of this phenol were higher in a range of 2.5-fold (line #1) to 4.6-fold (line #3) compared to EV. In lines #4-6, the levels of phenol no. 1 detected at RT of 5.3 min increased up to 2.8-fold, and phenol no. 4 at a RT of 12.5 min increased up to 7.2-fold compared to EV. Phenol no. 8 at a RT of 20.7 min increased only in lines #4 and #6 by 8.3-fold and 8.9-fold, respectively, compared to EV (Supplementary Figure S6).


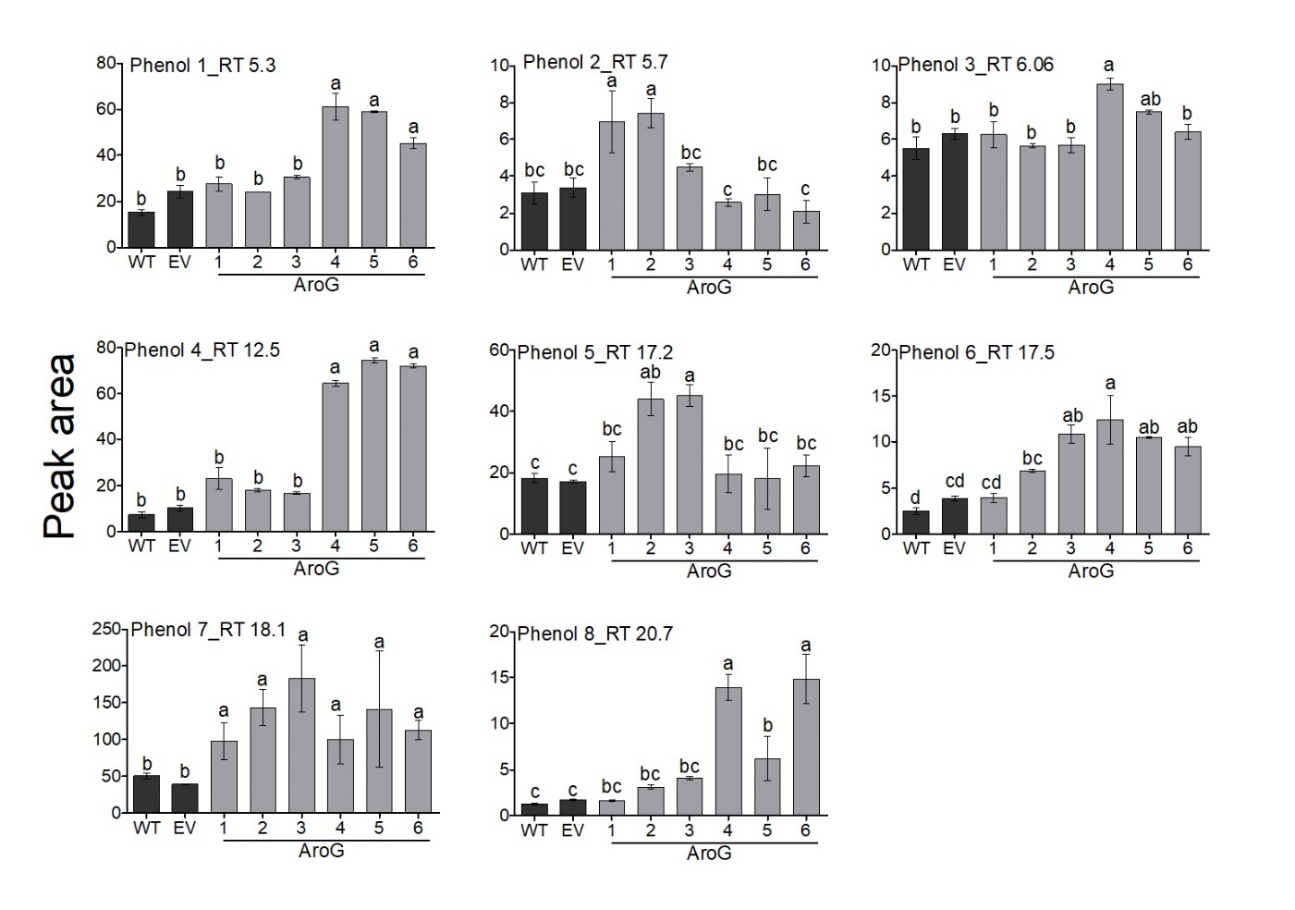


**FIGURE S6: Phenolic compounds found in leaves of AroG lines #1-6 and in EV/WT plants as measured at a wavelength of 280 nm using HPLC-DAD.** Data are presented as the mean ± SE of four biological repetitions. Different letters represent statistical significance (p≤0.05), which was determined using the Tukey-Kramer test.


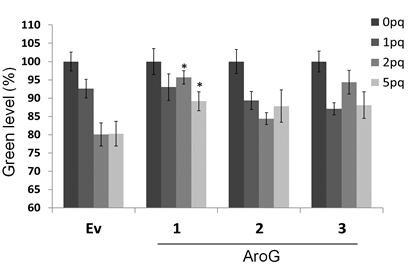


**A**

**FIGURE S7: The response of AroG lines to oxidative stress**. Eight mm discs (n=8-12) of transgenic AroG #1, #2, #3 and EV leaves (n=7) were exposed to different levels of in 0, 1, 2,5 µM methyl viologen (pq) for 20 h under 80-110 μE light conditions, and bleaching level was measured by ImageJ. Data are presented as a change (in %) from green level under the non-stress condition (0 pq) that was marked as 100%. Significance (p≤0.05) from EV at the same condition is marked by asterisks.

**
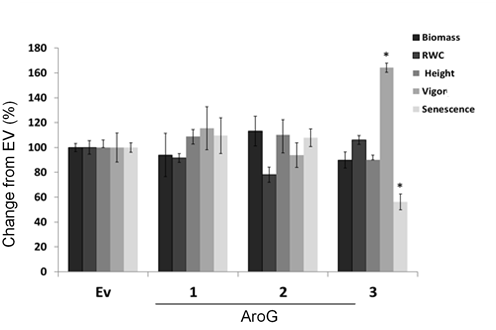
**

**FIGURE S8: The response of AroG lines to drought stress.** The plants were grown in a greenhouse as described in the Materials and Methods section. Parameters of senescence level and plant vigor were measured after the first drought period of 10 days, and parameters of height, dry biomass and relative water content (RWC) were measured at the end of the second drought period of 10 days. Data are presented as the mean ± SE of six biological replicates. Significance (p≤0.05) of each line compared to EV under the same conditions is marked by asterisks.


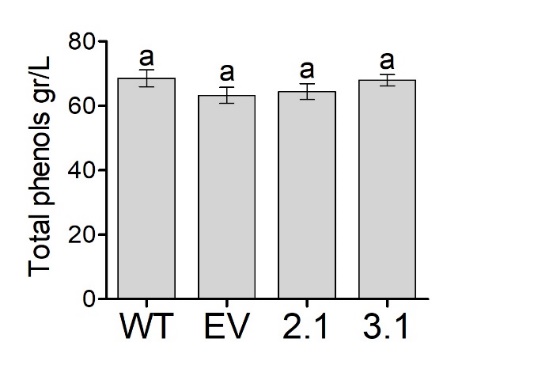


AroG

**FIGURE S9**: **Total polyphenol content (represented as g gallic acid equivalents per liter of water extract) in *P. aegyptiaca* grown on WT, EV and AroG lines.** Data are presented as the mean ± SE of *P. aegyptiaca* that were collected from five different pots. Different letters represent statistical significance (p≤0.05), which was determined using the Tukey-Kramer test.
